# Multiplex Immunofluorescent Batch Labeling of Marmoset Brain Sections

**DOI:** 10.1002/brb3.70308

**Published:** 2025-04-03

**Authors:** Daryan Chitsaz, Christopher D. Rowley, Nonthué A. Uccelli, Sarah Lefebvre, Andrea I. Krahn, Wolfgang E. Reintsch, Thomas M. Durcan, Christine L. Tardif, Timothy E. Kennedy

**Affiliations:** ^1^ Integrated Program in Neuroscience McGill University Montréal Quebec Canada; ^2^ Department of Neurology & Neurosurgery, Montreal Neurological Institute‐Hospital McGill University Montréal Quebec Canada; ^3^ Department of Physics & Astronomy McMaster University Hamilton Ontario Canada; ^4^ Cognitive Neuroscience Unit, Montreal Neurological Institute‐Hospital McGill University Montreal Quebec Canada; ^5^ The Neuro's Early Drug Discovery Unit (EDDU) McGill University Montreal Quebec Canada; ^6^ Department of Biomedical Engineering, Faculty of Medicine and Health Sciences McGill University Montréal Quebec Canada

**Keywords:** high‐throughput, histology, immunofluorescence, marmoset, myelin

## Abstract

**Purpose:**

The common marmoset is a small nonhuman primate that has emerged as a valuable animal model in neuroscience research. Accurate analysis of brain tissue is crucial to understand marmoset neurophysiology and to model neurodegenerative diseases. Many studies to date have complemented magnetic resonance imaging (MRI) with histochemical staining rather than immunofluorescent labeling, which can generate more informative and higher resolution images. There is a need for high‐throughput immunolabeling and imaging methodologies to generate resources for the burgeoning marmoset field, particularly brain histology atlases to display the organization of different cell types and other structures.

**Methods and Findings:**

Here, we have characterized a set of marmoset‐compatible fluorescent dyes and antibodies that label myelin, axons, dendrites, and the iron‐storage protein ferritin, and developed a batch‐style multiplex immunohistochemistry protocol to uniformly process large numbers of tissue slides for multiple cell‐type specific markers.

**Conclusion:**

We provide a practical guide for researchers interested in harnessing the potential of marmoset models to advance understanding of brain structure, function, and pathophysiology.

## Graphical abstract

To support high‐throughput immunolabeling and imaging methodologies for the burgeoning marmoset field, we characterize a set of marmoset‐compatible fluorescent dyes and antibodies and provide a practical batch‐style multiplex immunohistochemistry protocol for researchers interested in harnessing the potential of marmoset models to advance understanding of brain connectomics, function, and pathophysiology.

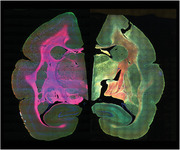

## 1 Introduction

The common marmoset (*Callithrix jacchus*) has become an increasingly popular model organism for neuroscientific analyses over the last two decades. Marmosets are small new‐world nonhuman primates (NHPs) that provide multiple advantages over other animal models. These include having more similar brain structures to humans than rodents (Schaeffer et al. 2022), exhibiting complex human‐like psychosocial behaviors (Miller et al. 2016), and reproducing and reaching maturity faster than other primate models (Tardif et al. 2003), together providing an invaluable platform for preclinical research. Marmosets have been used in a wide variety of neuroscientific studies ranging from tracking connectivity changes over their 10‐year lifespan to preclinical drug testing for Parkinson's disease (Hata et al. 2023; Yun, Ahn, and Kang 2015; Han, Powers, and Gabrielson 2022). To facilitate investigations like these, a number of initiatives have been launched to map the Marmoset brain (Okano 2021; Tokuno et al. 2009; Majka et al. 2016; Knauer et al. 2017; Kita et al. 2021; Shimogori et al. 2018; Atapour et al. 2019). The majority of these published atlases are magnetic resonance imaging (MRI)‐based with accompanying histochemical stains; two include immunolabeled neuronal markers (NeuN and four neuronal subtype markers) but use enzymatic histology rather than fluorescence (Tokuno et al. 2009; Atapour et al. 2019).

Classical histochemical methods typically broadly mark cellular structures, such as Nissl staining which binds RNA and DNA. In contrast, using immunolabeling, the specificity of antibody binding facilitates visualizing the distribution of virtually any protein. By employing fluorophores with different absorption/emission spectra, immunofluorescence provides the capacity to label multiple proteins of interest in a single tissue section, in combination with cell‐ or organelle‐specific markers. This allows multiple proteins, cellular subtypes, or structures to be labeled concurrently, revealing the relative distributions of differently labeled proteins and cells. The use of fluorophores provides a high signal‐to‐noise ratio that can be quantified, as the fluorescent signal intensity correlates with the amount of target protein. Immunofluorescence can also produce higher resolution and three‐dimensional images through confocal and super‐resolution imaging, compared to histochemistry, which typically utilizes widefield illumination and white‐light, such as bright‐field or phase contrast microscopy.

Optimizing immunofluorescence labeling for the marmoset central nervous system (CNS) has the potential to create new avenues to study their neurobiology, but presents technical challenges, especially when imaging across multiple brain regions is required. In particular, the epitopes that antibodies bind to are not necessarily conserved across species. Antibodies developed for research are often raised against rodent or human proteins and the epitope may not be conserved in homologous marmoset proteins. Methodological details such as fixative and buffer solutions can also alter antibody binding by modifying or masking epitopes. Further, polyclonal antibodies may exhibit different specificities between batches, even when purchased from the same company. Antibodies are also subject to degradation and depletion, meaning sequential labeling using the same solution of antibody may progressively become less saturated and dimmer. These challenges are compounded by the principle of “reduction” in animal care, which is of particular importance for NHPs. Marmosets are relatively rare and more expensive than typical rodent and nonmammalian models, further increasing the need to maximize the efficient use of their tissue.

Two advances are needed to facilitate whole brain‐scale marmoset immunofluorescence labeling. First, antibodies that work well in marmoset tissue should be identified, especially those that can function as markers for specific cell types or cellular structures, thereby saving investigators from having to search through costly trial and error. Ideally the antibodies should not require antigen retrieval, which may involve relatively rough pretreatment of the tissue, such as boiling in an acidic buffer (Im et al. 2019). While these extra manipulations can improve labeling and enable the use of more antibodies, they can also damage tissue and increase labeling variability between sections. Second, to make brain‐wide studies such as connectomic analyses possible, a high‐throughput batch‐style pipeline is needed so that all sections can be labeled homogeneously. Immunofluorescence labeling is often done on a perslide basis, however this can lead to increased variability of staining intensity, both within and between slides, due to the antibody being able to access the tissue to variable degrees.

**FIGURE 1:**
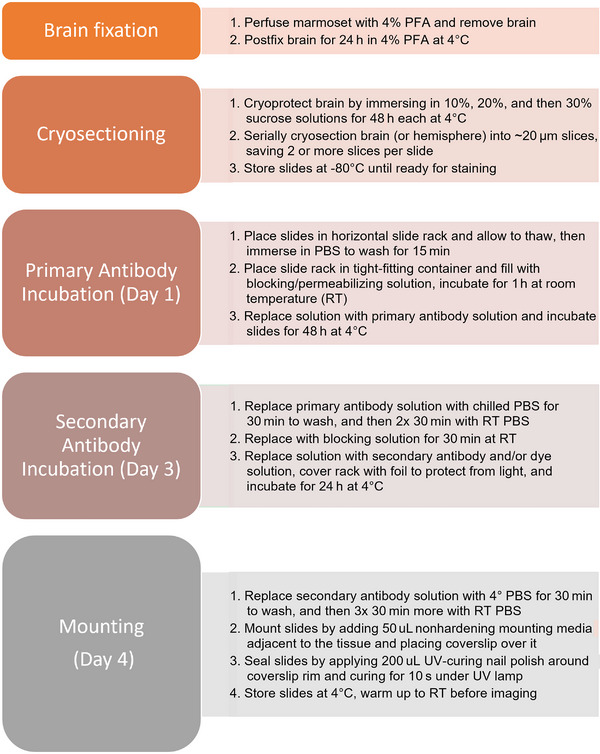
Batch‐style slide immunolabeling protocol. Brain fixation, cryopreservation, and sectioning takes at least 1 week after which slides can be stored until immunolabeling, which requires 4 days. All incubation steps should be carried out on a mechanical shaker. PBS, phosphate‐buffered saline; UV, ultraviolet.

Here, we describe a set of optimized antibodies and labels for fluorescent imaging, as well as a batch‐style adaptation of standard immunolabeling protocols to label brain structures on slide‐mounted brain tissue sections spanning one hemisphere of a marmoset brain (summarized in Figure 1). We labeled neuronal axons and dendrites, myelin proteins and lipids, cell nuclei, and ferritin, each of which are relevant to MRI techniques. We demonstrate the need for higher concentrations of permeabilizing detergent than what is typically used for mouse tissue immunolabeling to penetrate marmoset large white matter tracts. We also include advice to minimize errors and expedite the protocol, such as using ultraviolet (UV)‐curing nail polish to seal the slides. A further advantage of this mounting method is that it enables slides to be readily unmounted to restain the sections with minimal tissue damage. Aiming to optimize these techniques for a large‐scale marmoset brain mapping initiative (Rowley et al. 2023, 2021, 2023, 2021), we provide guidelines for batch immunolabeling and high‐throughput fluorescence imaging.

## 2 Methods and Results

### 2.1 Fixation and Tissue Sectioning

The study was conducted in accordance with the guidelines of the Canadian Council for Animal Care and approved by the McGill University Animal Care Committee. A 26‐month‐old female Common Marmoset was anaesthetized with an intramuscular injection of 20 mg/kg of ketamine (100 mg/mL), with 0.1 mL injections per site. Once anesthesia was induced, the marmoset was injected with 100 mg/kg of euthanyl (sodium pentobarbital 340 mg/mL; Bimeda). Reflexes were checked, the chest was rapidly opened, and a large gauge needle inserted into the left ventricle and the right atrium cut. Approximately 300 mL of heparinized saline was perfused via gravity. Following this, the same volume of 4% paraformaldehyde (PFA) was perfused, after which the brain was removed and postfixed for an additional 24 h by immersion in the same solution at 4°C. The brain was transferred to a 10% sucrose solution in phosphate‐buffered saline (PBS) for 2 days until it reached osmotic equilibrium and sank, after which it was transferred through solutions of 20% and 30% sucrose for an additional 2 days each. The right hemisphere was removed and stored, and 20 µm thick sections were cut from the left hemisphere using a cryostat (Leica SM2000R). Sections were mounted directly onto 25 × 75 mm Superfrost Plus microscope slides (Fisher) with 1–3 sections per slide depending on the size of the section. The use of Superfrost slides, which are precoated, prevents sections from detaching. Alternatively, conventional glass slides coated with gelatin could be used. Slides were stored in a slide box at −80°C until labeling.

### 2.2 Antibody Screening

A major goal of this study was to identify antibodies targeting proteins in marmoset brain tissue that label myelin, neuronal axons and dendrites, and the protein ferritin. By identifying such antibodies and characterizing their use, we aim to facilitate correlating immunohistochemical signals with high‐resolution MRI studies of the structure of the marmoset CNS. In particular, by labeling myelinated and unmyelinated axons and dendrites we sought to provide a ground‐truth for anatomical MRI scans. We targeted Myelin Basic Protein (MBP) to label myelin (Sternberger et al. 1978), β(III)‐tubulin for axons (Alexander et al. 1991), Microtubule‐Associated Protein 2 (MAP2) for dendrites (Peng, Binder, and Black 1986), and the MRI relevant protein ferritin (Fukunaga et al. 2010), the primary intracellular storage molecule for iron. To identify well‐validated antibodies for a given organism and application, we are typically guided by databases such as www.benchsci.com and YCharOS (Kahn, Virk, and McPherson 2023). A candidate antibody is then tested on sections of brain tissue to determine the ideal antibody concentration to be used. Multiple antibodies are then tested together to ensure that they and the associated secondary antibodies provide specific signals without cross‐reactivity. However, few antibodies reported in the literature have been used on marmoset tissue, and little “extra” marmoset tissue was available for us to perform antibody testing, necessitating a modified strategy.

**FIGURE 2:**
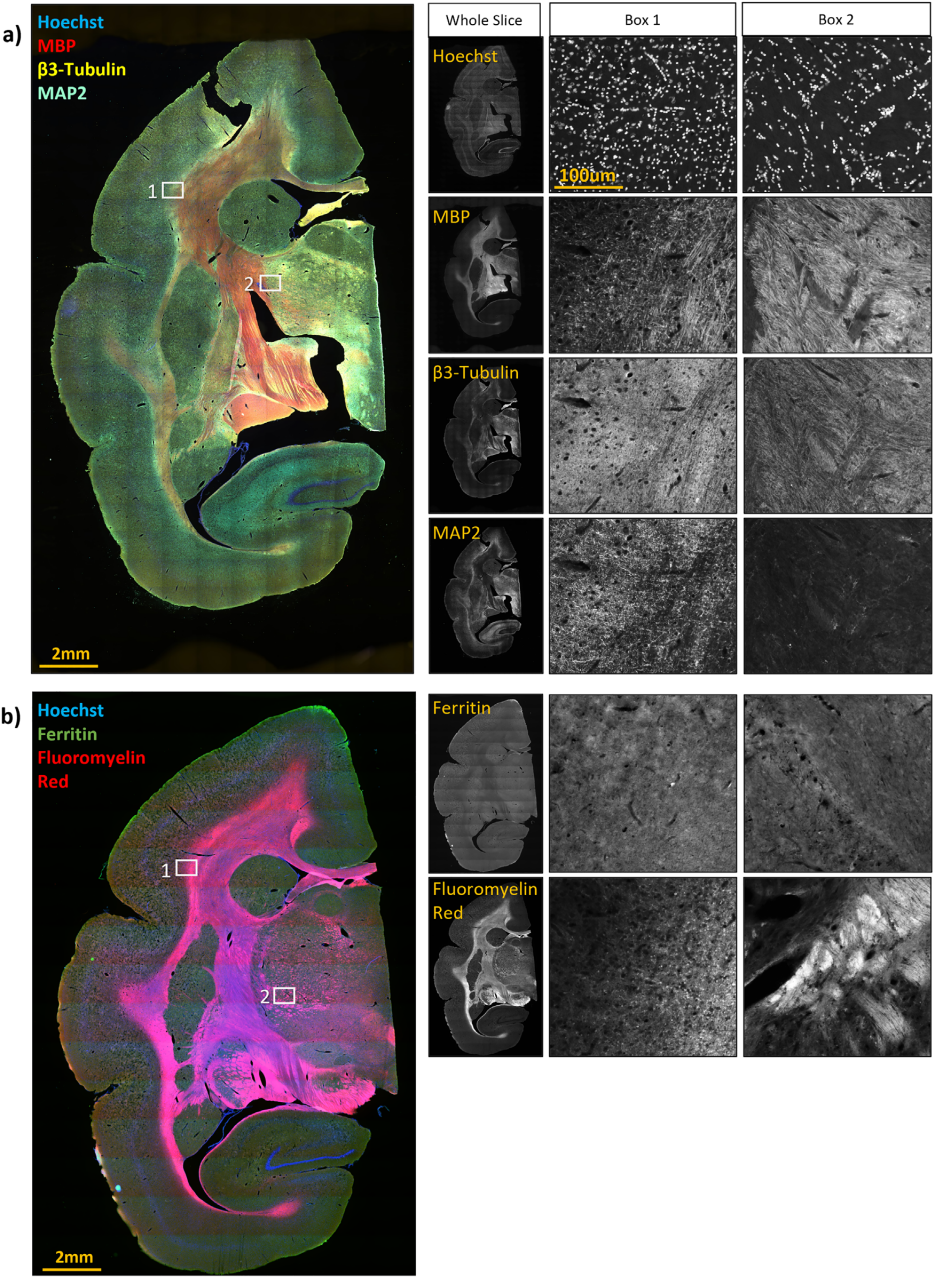
Photomicrographs of multiplexed immunolabeling on hemisections of marmoset brain. The middle and rightmost columns present magnified images of the boxed regions on the multicolor images. (A) Hoechst dye with immunolabeling for Myelin Basic Protein (MBP), β3‐tubulin, and Microtubule‐Associated Protein 2 (MAP2). (B) Shows α‐ferritin labeling and staining with the dye Fluoromyelin Red.

**FIGURE 3:**
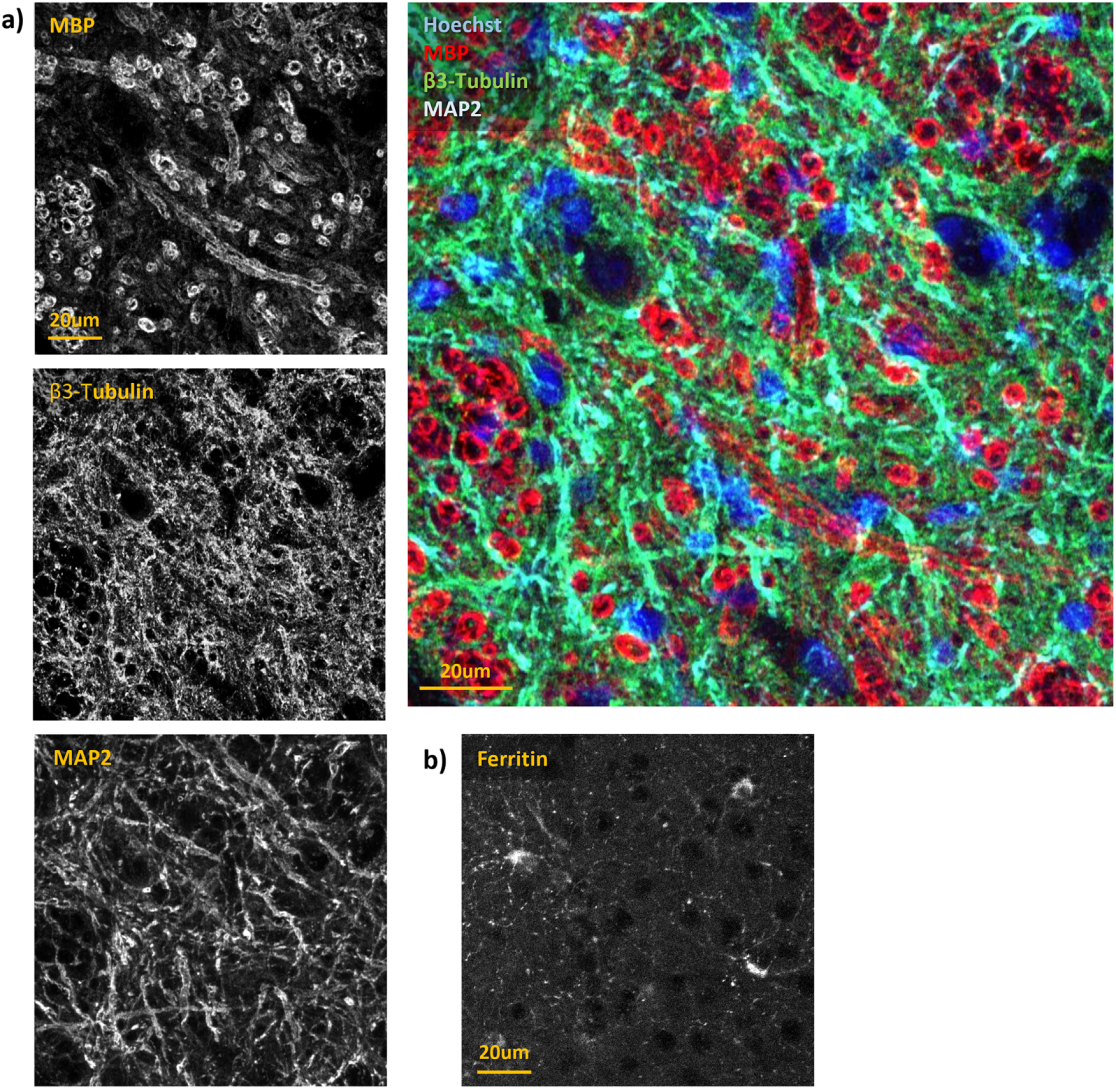
Higher magnification confocal microscope images of the distribution of labeling obtained for each of the four antibodies. (A) Multichannel images show myelin sheaths immunolabeled for Myelin Basic Protein (MBP), β3‐tubulin labeling in axons, and Microtubule‐Associated Protein 2 (MAP2) labeling in dendrites, with an enlarged merged image. (B) Ferritin immunolabeling is highly punctate and concentrated in glia. The ferritin image is from a different tissue section from that shown in (A).

We identified a set of candidate antibodies that had been validated using both human and mouse tissue, indicating conservation of the binding epitope between human and rodents, and reasoned that these had a good chance of binding homologous marmoset proteins (all tested antibodies are listed in Table 1). Rather than testing them individually in marmoset, we first tested them together in mouse, initially at the lowest concentration recommended by the manufacturer. It should be noted that in our experience these recommended concentrations are typically higher than necessary. We then tested the antibody combinations together in marmoset tissue to ensure that they labeled the expected targets and did not show signs of off‐target binding. A number of the antibodies tested, listed in Table 1, did not produce the expected labeling. Fluoromyelin Red and Hoechst 33342, two non‐antibody dyes that stain lipids in myelin and cell nuclei, respectively (Kilgore and Janes 2004; Weisblum and Haenssler 1974; Monsma and Brown 2012) were used at the concentrations recommended by manufacturers. The optimized set of antibodies and dyes along with their concentrations and other considerations are presented in Table 2, with representative whole‐slice widefield fluorescent microscopy images presented in Figure 2. Figure 3 shows higher resolution confocal images of MBP, β3‐tubulin, and MAP2‐labeled sections, visualizing labeling for myelin and neuronal axons and dendrites, as well as ferritin‐labeled tissue with signal clustered around glia, consistent with relatively high expression by oligodendrocytes (Friedrich et al. 2021; Todorich et al. 2009).

**TABLE 1:** Antibodies that did not produce the expected labeling.

Antigen	Target structure	Species/clonality	Company	Product	Result
MAP2	Dendrites	Chick	Genetex	GTX85455	No labeling
MAP2	Dendrites	Chick	Genetex	GTX82661	No labeling
MAP2	Dendrites	Rabbit	Abcam	Ab32454	Weak labeling
MAP2	Dendrites	Chick	EncorBio	CPCA‐MAP2	No labeling
MBP	Myelin	Chick	Aves	MBP	No labeling
MBP	Myelin	Rat	Millipore	MAB386	Nonspecific
NF‐H	Axons	Mouse monoclonal IgM	Biolegend	835704	No labeling
NF‐H (Smi31)	Axons	Mouse IgG1	Biolegend	801602	No labeling
NF‐M	Axons	Chick	Aves	NFM	Nonspecific
PLP	Myelin	Chick	Aves	PLP	Weak labeling
TUBB3 (Tuj1)	Axons	Mouse monoclonal IgG2a	Biolegend	801201	Weak labeling

Abbreviations: MAP2, Microtubule‐Associated Protein 2; MBP, Myelin Basic Protein; NF, Neurofilament; PLP, proteolipid protein; TUBB3, tubulin beta class III.

All antibodies were polyclonal unless marked otherwise.

**TABLE 2:** Fluorescent dyes, and primary and secondary antibodies, optimized for marmoset brain sections.

Dye	Dilution	Product	Notes
Hoechst 33342	1:2000	H3469	
Fluoromyelin Red	1:300	F34652	Sensitive to Triton X‐100

Primary antibodies					
Antigen	Species	Dilution	Company	Product	Notes
β‐(III) tubulin	Rabbit (polyclonal)	1:2000	BioLegend	802001 (formerly PRB‐435P)	
MBP	Rat (monoclonal)	1:1000	Millipore Sigma	MAB386	Requires high Triton X‐100 concentration (1.0%)
MAP2	Chicken (polyclonal)	1:2000	Abcam	Ab5392	Sensitive to Triton X‐100 concentration (above 0.5%)
Ferritin	Rabbit (polyclonal)	1:1000	Millipore Sigma	F5012	

Secondary antibodies (all raised in goat, used at 1:1000, from ThermoFisher)		
Excitation wavelength	Target species	Product
488	Rabbit	A‐11008
555	Rat	A‐21434
647	Chick	A‐21449

Abbreviations: MAP2, Microtubule‐Associated Protein 2; MBP, Myelin Basic Protein. RRIDs: AB2_564645, tubulin; AB_94975, MBP; AB_2138153, MAP2; AB_259622, Ferritin; AB_143165, Rabbit 488; AB_2535855, Rat 555; AB_2535866, Chick 647.

Dyes were applied along with secondary antibodies.

**FIGURE 4:**
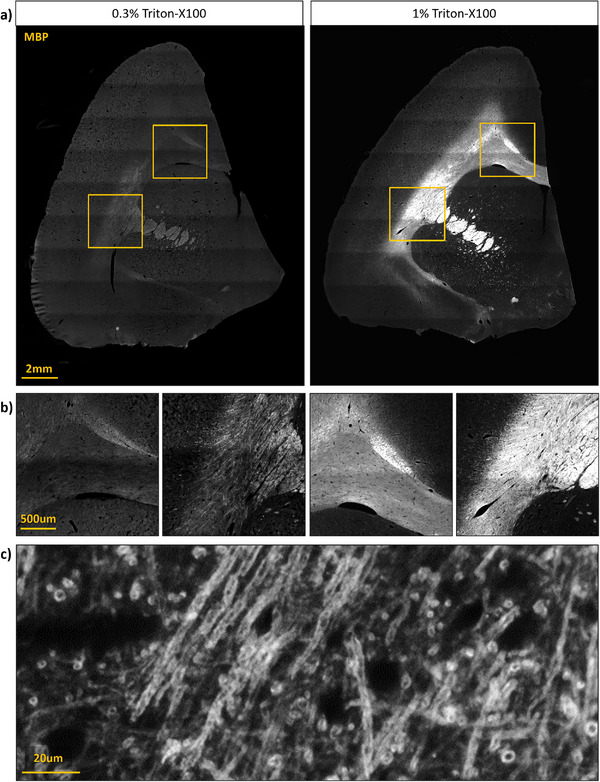
Comparing permeabilization conditions for Myelin Basic Protein (MBP) immunolabeling. (A) Marmoset brain hemisection permeabilized with 0.3% Triton X‐100 on the left, and then after relabeling with 1.0% Triton X‐100 permeabilization. (B) Left and right images present magnified images of the top and bottom boxes, respectively, revealing brighter and more detailed MBP labeling. (C) Higher magnification image of myelinated axons in the corpus callosum, from a separate section permeabilized with 1.0% Triton X‐100, in which individual myelin sheaths are clearly resolved.

A significant challenge we encountered was tissue permeabilization. Antibodies are relatively large molecules that cannot diffuse through cell membranes, so fixed tissue sections are typically permeabilized by adding a detergent solution such as Triton X‐100 diluted in PBS, to allow antibody penetration. High levels of detergent are required to label structures embedded in multiple layers of membrane, such as myelin, but detergents can also disrupt protein complexes, impair other labeling, and increase autofluorescence (Suah, Ahmad, and Mhamod 2017). We found that detergent concentration levels that are sufficient to permeabilize mouse brain myelin (0.3% Triton X‐100) were too low to provide evenly labeled MBP in the thicker corpus callosum of the marmoset, though tracts that were cut cross‐sectionally were labeled relatively well, as in these cases the antibody did not need to penetrate through layers of lipids. We found that a 1.0% solution of Triton X‐100 permeabilized the tissue sufficiently to result in clearly immunolabeled MBP (Figure 4).

### 2.3 Batch Immunofluorescent Labeling

The batch immunolabeling protocol is summarized in Figure 1. In preparation for a brain mapping initiative, we aimed to maximize labeling homogeneity by processing up to 60 slides simultaneously. All immunohistochemical labeling and washes were performed via simultaneous immersion of all samples together to promote homogeneous staining. Slides were removed from storage in a −80°C freezer and placed on a bench for 5 min at room temperature (RT, 20–23°C), allowing them to thaw but not fully dry. Slides were then loaded into a 60‐slide metal rack (TedPella #21058) which was immersed in a close‐fitting container filled with PBS at RT to wash away any residual cryosectioning solution, with the top covered in Parafilm to prevent evaporation or spilling. The PBS was then replaced and the slides incubated at RT for 1 h in a permeabilizing‐and‐blocking solution composed of PBS with 3% heat‐inactivated horse serum and 3% bovine serum albumin (BSA; w/v), and varying concentrations of Triton‐X100 (see Table 2 for final optimized Triton X‐100 concentrations for each antibody or dye). The permeabilizing‐and‐blocking solution was then replaced by the same solution with primary antibodies added (see Table 2 for antibodies and concentrations used). We required 400 mL of solution to cover the rack of 60 slides such that the tissue was entirely immersed. Slides were then incubated for 48 h in the primary antibody‐containing solution, at 4°C, with gentle agitation at 30 rpm on a rotary shaker (G2 Gyratory Shaker, New Brunswick Scientific Co.). The container was covered in Parafilm to prevent evaporation. The solution was then removed and the slides washed 2 × 30 min in PBS, and then returned for 1 h to the same blocking‐and‐permeabilizing solution at RT. The initial PBS wash was done using chilled PBS at 4°C so that the cold slides that had been incubated with primary antibody for 48 h at 4°C did not undergo a dramatic temperature shift. Subsequent washes were carried out using solutions at RT. The slides were then immersed in the blocking‐and‐permeabilizing solution with secondary antibodies added, and in some cases with Hoechst dye or Fluoromyelin Red dye added. The container was once again sealed with Parafilm and covered with aluminum foil to protect the labels from photobleaching, and incubated at 4°C for 24 h. Slides were then washed once again using PBS at 4°C for 30 min, then 2 × 30 min with PBS at RT, while continuing to protect from light. Labeling solutions were stored at 4°C, with the tubes wrapped in aluminum foil to protect against light, with 0.05% sodium azide added as a preservative. In our experience, the antibody‐containing solutions could be used to label additional slides up to at least 6 months later.

### 2.4 Mounting Labeled Tissue Sections

After the final PBS wash, one slide at a time was removed, dipped briefly in milli‐Q H_2_O, had its edge tapped dry on a paper towel to wick away residual liquid, and ∼50 µL of nonhardening mounting media (Fluoromount‐G, Fisher) was then applied over the tissue sections using a cut P200 pipette tip. Nonhardening media preserve tissue morphology better than hardening varieties which can flatten samples as they cure but requires sealing the edges of the coverslip during mounting, which creates a challenge for high‐throughput applications in which dozens or hundreds of slides need to be mounted concurrently. To overcome this, we previously developed a technique (Kennedy et al. 2023) that applies commercially available UV‐curing nail polish (Complete Salon Manicure Fast Dry Top Coat, Sally Hansen) to seal coverslips rather than conventional slow air‐drying nail polish. After applying the mounting media, a 22 × 50 mm coverslip (Fisherbrand) was placed overtop and sealed by dispensing UV‐curing nail polish around the edge of the coverslip using a cut p200 pipette tip, which was then rapidly hardened using a nail polish curing chamber (T5 72 W LED UV Nail Lamp, Amazon) for 10 s. This brief UV treatment was previously demonstrated to not detectably fade Hoechst dye or Alexa488 fluorophores (Kennedy et al. 2023). Slides could then be placed directly in a slide storage box or imaged immediately. Sealed slides were stored at 4°C until imaging.

### 2.5 Tissue Relabeling

To better permeabilize the tissue after poor MBP immunolabeling, we took advantage of the UV‐curing gel polish which can be removed more easily than conventional nail polish. By sliding a razor blade under the corner of the nail polish seal, we were able to pull the rest off cleanly without causing shearing between the microscope slide and coverglass. The slide was then immersed in PBS at RT in a 50 mL falcon tube for 1–2 min to dissolve the mounting media, after which the slide could gently be pulled out, leaving the coverglass in the falcon tube without damaging the tissue. The slide was immediately relabeled using the same conditions and antibody concentration (Table 2), but with 1.0% Triton X‐100 instead of the original 0.3% (Figure 4).

### 2.6 Imaging

The images presented in Figure 2 were acquired with a Zeiss AxioObserver Z1 inverted microscope with a 0.4 NA 10× objective using a Colibri 2 LED lightsource and Zen Blue 3.0 software (Zeiss). Approximately 15 × 15 tiles were acquired for each image, which were then stitched into a composite image with Zen software. Higher resolution images shown in Figure 3 were acquired with an LSM880 confocal microscope and 0.8 NA 20× objective using Zen Black 2.1 software (Zeiss). To generate the composite image shown in Figure 4, whole‐slide imaging was performed using an ImageXpress High‐Content Imaging System with a 0.4 NA 10× objective (Molecular Devices). Image tiles were acquired for each section using a custom slide scanning protocol macro (Molecular Devices, MetaXPress v6.7.2). Focal points were acquired manually for each slide. Sixteen‐bit images were acquired at a resolution of 0.67 µm/pixel. Each three‐ or four‐channel image consisted of 10–20 tiles squared (between 100 to 400 tiles total), taking 5–20 min to acquire and using over 1 GB of storage. Image tiles were exported from MetaXPress as TIFF files, and then sorted into folders for each channel and stitched with the Grid/Collection Stitching plugin with a custom ImageJ macro in FIJI (Schindelin et al. 2012; Preibisch, Saalfeld, and Tomancak 2009).

### 2.7 Antibody Characterization

The specificity of immunoreactivity was assessed by comparing the distribution of labeling in marmoset tissue to mouse, in which all antibodies had been previously characterized in the literature. Polyclonal antibodies against beta(III)‐tubulin (Biolegend TUBB3) were raised against rat brain‐derived tubulin and recognize an epitope within the last 15 C‐terminal residues. Western blot and immunohistochemical analyses have demonstrated neuronal‐specific immunoreactivity marking axons (Lee, Rebhun, and Frankfurter 1990; Ambasudhan et al. 2011). Polyclonal antibodies against MAP2 (Abcam ab5392) were raised against sequences between amino acids 235–1588 of human MAP2 protein. Immunocytochemical analyses revealed somato‐dendritic labeling, and positive immunoreactivity on western blot analyses of human neuronal homogenates, but not undifferentiated precursors (Park et al. 2022). Monoclonal MBP antibody (EMD Millipore MAB386) recognizes an epitope between amino acids 82–87 that is shared by the four human MBP isoforms expressed in the CNS. Reduced labeling intensity was detected on western blots and immunohistochemical analyses of hypomyelinated mice (Lin et al. 2017). Polyclonal antibodies against ferritin (Sigma F5012) were raised against the human ferritin heavy chain, with western blot analyses and immunofluorescence demonstrating specific labeling of treated cells in an iron‐loading assay (Healy et al. 2016).

## 3 Discussion

Here, we identify four antibodies for immunofluorescence and two fluorescent stains that can be used with PFA‐fixed marmoset tissue. To identify these functional antibodies, we screened 11 other antibodies which failed to effectively label marmoset tissue (Table 1), highlighting the importance of publishing antibodies that are incompatible with this species and method of sample preparation. We also outline a modified immunostaining protocol that employs larger volumes of solution in order to uniformly label dozens of slide‐mounted tissue sections concurrently, to aid high‐throughput initiatives such as those required for expression atlases and connectome tracing. We additionally describe a method to relabel already mounted tissue sections that have been sealed with commercially available UV‐curing nail polish (Kennedy et al. 2023), which allows for different labeling conditions to be applied sequentially, reducing the need for additional sections during optimization and providing an avenue to overcome mistakes made when labeling scarce tissue.

Several marmoset brain mapping initiatives employing histology have been reported to date. The first publicly available digital dataset was an atlas (Tokuno et al. 2009) that included histological stains like Nissl, which marks nucleic acids in neuronal and glial cell bodies, and tissue sections that were individually labeled for one of four neuronal markers and visualized using enzymatic immunohistology, marking neurofilament, tyrosine hydroxylase, parvalbumin, and calbindin (Tokuno et al. 2009). This atlas was used by Paxino et al. (2012) to generate a parcellation scheme of 116 cytoarchitectural areas. Majka et al. (2020, 2016) combined Nissl staining with Adeno Associated Virus (AAV) based tract tracing to create a Marmoset Connectivity Atlas (Majka et al. 2020, 2016), providing high‐resolution connectivity data for projections neurons in five areas of the cortex. Other atlases have combined Nissl and silver myelin histochemical staining with mass spectrometry‐based metallomic analysis (Knauer et al. 2017), MRI (T1, T2, and dTI; Schaeffer et al. 2022; Lin et al. 2019; Majka et al. 2021), or gene expression in developing and adult animals via in situ hybridization (Kita et al. 2021; Shimogori et al. 2018). A stereological analysis of neuronal distribution was also reported from sections immunolabeled for NeuN, which marks neuronal nuclei (Atapour et al. 2019).

Each brain atlas provides a powerful resource to understand marmoset brain development, connectivity, and anatomy, but multiplex immunolabeling can provide further insight. Specifically, the existing atlases provide no information on glial cell distribution or connectivity for most brain regions. The published atlases also only employ one stain or immunolabel per section, while multiplex labeling allows multiple proteins or cell markers to be visualized simultaneously, for example, displaying how disease‐associated proteins accumulate in differently labeled neuronal populations. Multiplexing can reveal qualitative and quantitative relationships between labels while reducing the number of samples that need to be processed and imaged, reducing the number of animals required for a complex study. Antibody labeling can be combined with other imaging techniques such as Fluoromyelin dye (Monsma and Brown 2012) or genetically encoded fluorophore labeling. Immunofluorescence labeling can also generate higher resolution images to better capture neural cells’ three‐dimensional morphologies, and precisely localize labeled proteins. In contrast, enzymatic reactions used by histochemical techniques often produce a colored stain that fills cells but confound subcellular localization. Fluorescence microscopy can also label a wide variety of biologically relevant targets including metabolites, specific DNA or RNA sequences, and post‐translational modifications, in addition to virtually any protein.

Immunofluorescence of NHP tissue does come with several limitations, chief of which is the challenge of finding validated monoclonal antibodies that specifically bind marmoset proteins (without cross‐reacting with other labels) for a given fixation protocol. Because monoclonal antibodies are primarily made in mouse and rabbit, multiplexing with three or more fluorophores often requires utilizing a polyclonal antibody from another species which adds additional batch‐to‐batch variability. Antibodies are also particularly costly compared to classical histochemical stains. This can be partially mitigated by reusing antibody‐containing solutions, though different batches may not be directly comparable as the antibodies will be depleted and may slowly degenerate over time even when optimally stored. An additional challenge is the possible requirement for different concentrations of detergent for different antibodies. Antibodies are proteins, some of which are more stable than others, and proteins can be disrupted by high concentrations of detergents, like Triton‐X100, while others require high concentrations to sufficiently permeabilize the tissue, such as the labeling for MBP carried out here and potentially for other myelin‐associated proteins. The properties of the sample may also affect immunofluorescence labeling—different fixatives may generate different chemical cross‐links, potentially masking or chemically modifying epitopes on the target protein of interest. Thicker or more dense tissue sections may require longer incubation times to allow antibodies to diffuse through the tissue. One option that may improve label penetration is to store and stain tissue as floating sections in the wells of a tissue culture plate rather than mounted on slides (Potts, Coppotelli, and Ross 2020). This configuration allows antibodies to diffuse into the tissue from all sides and may lower cost by reducing the amount of solution needed, especially if multiple sections can be stored in each well. However, floating sections can be less practical than staining slide‐mounted tissue when large numbers of slices are needed, and when larger brains are sectioned. The sequence of sections of tissue becomes mixed when stored as free‐floating sections in the same well, and large sections of fragile brain tissue may be more likely to become damaged, deformed, or fragmented when changing solutions or subsequently mounting the labeled sections onto a slide.

In addition to technical issues that may arise during tissue immunolabeling, high‐throughput fluorescence microscopy also presents challenges. Adult marmoset brain sections can be large enough to necessitate acquiring hundreds of small image tiles that then must be stitched together, requiring automated microscopes. Many options exist for whole‐slide imaging ranging from standard laboratory microscopes with motorized stages to dedicated slide scanners, each of which has strengths and weaknesses, but some general points are worth considering. First, using an imaging system with a large field of view will reduce the number of tiles that need to be acquired for each section. This will shorten imaging time and, importantly, mitigate refocusing and image tile stitching artifacts. Based on the resolution required, the appropriate objective is chosen to achieve the desired resolution while maximizing image size—in our case, a 0.4 NA 10× objective was sufficient to resolve individual myelin sheaths wrapping cortical axons which are approximately 1 um diameter (Figures 3 and 4). Second the microscope slide stage should be inspected prior to mounting the tissue sections on the slides to determine if it is appropriate. Upright microscopes with the slides sitting face‐up rarely pose problems, however some slide holders for inverted microscopes have ledges that may interfere with larger coverslips, causing the slide to sit unevenly depending on where the coverslip is located and the thickness of the coverslip sealant that was used. It is essential that the slide sit flat during imaging to maintain focus while collecting images to be tiled. Furthermore, not all imaging systems have built‐in digital image stitching capabilities. Notably, the MetaXPress HCI software used here, lacked sufficient memory to stitch the large number of tiles needed for a single marmoset section, necessitating third party software and time‐consuming workarounds. Finally, file transfer and storage can be a challenge with high‐resolution multichannel images. In our dataset, individual whole‐section tiled images with four channels and a 0.67 µm/pixel resolution were up to 5 GB each, making cloud storage expensive and prohibitively slow.

Marmosets have tremendous potential to address numerous challenges in neuroscience. This will be expedited by sharing optimized protocols, including identifying reagents that do not work. Establishing reliable methods for immunofluorescence labeling will help characterize marmoset brain anatomy as well as pave the way for more targeted studies into specific circuits and regions, ultimately contributing to advancements and therapeutic interventions for neurological conditions.

## Author Contributions

Daryan Chitsaz conceived and optimized the immunolabeling protocol and wrote the original manuscript. Daryan Chitsaz, Christopher D. Rowley, and Nonthué A. Uccelli performed immunolabeling and imaging, and manuscript editing. Sarah Lefebvre fixed and cryosectioned samples. Andrea I. Krahn and Wolfgang E. Reintsch provided technical imaging assistance. Christine L. Tardif, Timothy E. Kennedy, and Thomas M. Durcan provided supervision, editing, resources, and funding acquisition.

## Ethics Statement

The animal tissue used for this study was obtained in accordance with the guidelines of the Canadian Council for Animal Care and approved by the McGill University Animal Care Committee.

## Conflicts of Interest

The authors declare no conflicts of interest.

### Peer Review

The peer review history for this article is available at https://publons.com/publon/10.1002/brb3.70308.

## Data Availability

All images used can be made available upon request from the authors.

## References

[brb370308-bib-0001] Alexander, J. E. , D. F. Hunt , M. K. Lee , et al. 1991. “Characterization of Posttranslational Modifications in Neuron‐Specific Class III Beta‐Tubulin by Mass Spectrometry.” PNAS 88, no. 11: 4685–4689. 2052551 10.1073/pnas.88.11.4685 PMC51730

[brb370308-bib-0002] Ambasudhan, R. , M. Talantova , R. Coleman , et al. 2011. “Direct Reprogramming of Adult Human Fibroblasts to Functional Neurons Under Defined Conditions.” Cell Stem Cell 9, no. 2: 113–118. 21802386 10.1016/j.stem.2011.07.002 PMC4567246

[brb370308-bib-0003] Atapour, N. , P. Majka , I. H. Wolkowicz , D. Malamanova , K. H. Worthy , and M. G. P. Rosa . 2019. “Neuronal Distribution Across the Cerebral Cortex of the Marmoset Monkey (*Callithrix jacchus*).” Cerebral Cortex (New York, N.Y.: 1991) 29, no. 9: 3836–3863. 30357325 10.1093/cercor/bhy263

[brb370308-bib-0004] Friedrich, I. , K. Reimann , S. Jankuhn , et al. 2021. “Cell Specific Quantitative Iron Mapping on Brain Slices by Immuno‐µPIXE in Healthy Elderly and Parkinson's Disease.” Acta Neuropathologica Communications 9, no. 1: 47. 33752749 10.1186/s40478-021-01145-2 PMC7986300

[brb370308-bib-0005] Fukunaga, M. , T. Q. Li , P. van Gelderen , et al. 2010. “Layer‐Specific Variation of Iron Content in Cerebral Cortex as a Source of MRI Contrast.” PNAS 107, no. 8: 3834–3839. 20133720 10.1073/pnas.0911177107 PMC2840419

[brb370308-bib-0006] Han, H. J. , S. J. Powers , and K. L. Gabrielson . 2022. “The Common Marmoset‐Biomedical Research Animal Model Applications and Common Spontaneous Diseases.” Toxicologic Pathology 50, no. 5: 628–637. 35535728 10.1177/01926233221095449 PMC9310150

[brb370308-bib-0007] Hata, J. , K. Nakae , H. Tsukada , et al. 2023. “Multi‐Modal Brain Magnetic Resonance Imaging Database Covering Marmosets With a Wide Age Range.” Scientific Data 10, no. 1: 221. 37105968 10.1038/s41597-023-02121-2 PMC10250358

[brb370308-bib-0008] Healy, S. , J. McMahon , P. Owens , and U FitzGerald . 2016. “Significant Glial Alterations in Response to Iron Loading in a Novel Organotypic Hippocampal Slice Culture Model.” Scientific Reports 6: 36410. 27808258 10.1038/srep36410 PMC5093415

[brb370308-bib-0009] Im, K. , S. Mareninov , M. F. P. Diaz , and W. H Yong . 2019. “An Introduction to Performing Immunofluorescence Staining.” Methods in Molecular Biology 1897: 299–311. 30539454 10.1007/978-1-4939-8935-5_26 PMC6918834

[brb370308-bib-0010] Kahn, R. A. , H. S. Virk , and P. S McPherson . 2023. “Heed a Decade of Calls for Antibody Validation.” Nature 620, no. 7974: 492. 10.1038/d41586-023-02566-w 37582877

[brb370308-bib-0011] Kennedy, G. , D. Chitsaz , J. F. Madranges , M. Peştemalciyan , A. F. Sadikot , and T. E. Kennedy . 2023. “Gel Nail Polish as an Alternative to Traditional Coverslip Sealants: A Quick Solution to a Sticky Situation.” MethodsX 11: 102256. 37383626 10.1016/j.mex.2023.102256 PMC10293718

[brb370308-bib-0012] Kilgore, J. , and M Janes . 2004. A Novel Myelin Labeling Technique for Fluorescence Microscopy of Brain Sections. Society for Neuroscience.

[brb370308-bib-0013] Kita, Y. , H. Nishibe , Y. Wang , et al. 2021. “Cellular‐Resolution Gene Expression Profiling in the Neonatal Marmoset Brain Reveals Dynamic Species‐ and Region‐Specific Differences.” PNAS 118, no. 18: e2020125118. 33903237 10.1073/pnas.2020125118 PMC8106353

[brb370308-bib-0014] Knauer, B. , P. Majka , K. J. Watkins , et al. 2017. “Whole‐Brain Metallomic Analysis of the Common Marmoset (*Callithrix jacchus*).” Metallomics 9, no. 4: 411–423. 28246661 10.1039/c7mt00012j

[brb370308-bib-0015] Lee, M. K. , L. I. Rebhun , and A. Frankfurter . 1990. “Posttranslational Modification of Class III Beta‐Tubulin.” PNAS 87, no. 18: 7195–7199. 2402501 10.1073/pnas.87.18.7195 PMC54710

[brb370308-bib-0016] Lin, J. P. , Y. A. Mironova , P. Shrager , and R. J. Giger . 2017. “LRP1 Regulates Peroxisome Biogenesis and Cholesterol Homeostasis in Oligodendrocytes and Is Required for Proper CNS Myelin Development and Repair.” Nave K. A., editor. Elife 6: e30498. 29251594 10.7554/eLife.30498 PMC5752207

[brb370308-bib-0017] Lin, M. K. , Y. S. Takahashi , B. X. Huo , et al. 2019. “A High‐Throughput Neurohistological Pipeline for Brain‐Wide Mesoscale Connectivity Mapping of the Common Marmoset.” Helmstaedter M., Marder E., Vidyasagar T. R., Helmstaedter M., editors. Elife 8: e40042. 30720427 10.7554/eLife.40042 PMC6384052

[brb370308-bib-0018] Majka, P. , S. Bai , S. Bakola , et al. 2020. “Open Access Resource for Cellular‐Resolution Analyses of Corticocortical Connectivity in the Marmoset Monkey.” Nature Communications 11, no. 1: 1133. 10.1038/s41467-020-14858-0 PMC7048793 32111833

[brb370308-bib-0019] Majka, P. , S. Bednarek , J. M. Chan , et al. 2021. “Histology‐Based Average Template of the Marmoset Cortex With Probabilistic Localization of Cytoarchitectural Areas.” Neuroimage 226: 117625. 33301940 10.1016/j.neuroimage.2020.117625

[brb370308-bib-0020] Majka, P. , T. A. Chaplin , H. H. Yu , et al. 2016. “Towards a Comprehensive Atlas of Cortical Connections in a Primate Brain: Mapping Tracer Injection Studies of the Common Marmoset Into a Reference Digital Template.” Journal of Comparative Neurology 524, no. 11: 2161–2181. 27099164 10.1002/cne.24023 PMC4892968

[brb370308-bib-0021] Miller, C. T. , W. A. Freiwald , D. A. Leopold , J. F. Mitchell , A. C. Silva , and X. Wang . 2016. “Marmosets: A Neuroscientific Model of Human Social Behavior.” Neuron 90, no. 2: 219–233. 27100195 10.1016/j.neuron.2016.03.018 PMC4840471

[brb370308-bib-0022] Monsma, P. C. , and A Brown . 2012. “FluoroMyelin^TM^ Red Is a Bright, Photostable and Non‐Toxic Fluorescent Stain for Live Imaging of Myelin.” Journal of Neuroscience Methods 209, no. 2: 344–350. 22743799 10.1016/j.jneumeth.2012.06.015 PMC3429707

[brb370308-bib-0023] Okano, H. 2021. “Current Status of and Perspectives on the Application of Marmosets in Neurobiology.” Annual Review of Neuroscience 44, no. 1: 27–48. 10.1146/annurev-neuro-030520-101844 34236888

[brb370308-bib-0024] Park, S. Y. , D. S. Kim , H. M. Kim , et al. 2022. “Human Mesenchymal Stem Cell‐Derived Extracellular Vesicles Promote Neural Differentiation of Neural Progenitor Cells.” International Journal of Molecular Sciences 23, no. 13: 7047. 35806058 10.3390/ijms23137047 PMC9267053

[brb370308-bib-0025] Paxinos, G. , C. Watson , M. Petrides , M. Rosa , and H. Tokuno . 2012. “Scalable Brain Atlas. Marmoset—[Internet].” Accessed March 31, 2024. https://scalablebrainatlas.incf.org/marmoset/PWPRT12.

[brb370308-bib-0026] Peng, I. , L. I. Binder , and M. M. Black . 1986. “Biochemical and Immunological Analyses of Cytoskeletal Domains of Neurons.” Journal of Cell Biology 102, no. 1: 252–262. 3510221 10.1083/jcb.102.1.252 PMC2114054

[brb370308-bib-0027] Potts, E. M. , G. Coppotelli , and J. M. Ross . 2020. “Histological‐Based Stainings Using Free‐Floating Tissue Sections.” Journal of Visualized Experiments. Aug 25(162). 10.3791/61622. PMC7743918 32925894

[brb370308-bib-0028] Preibisch, S. , S. Saalfeld , and P. Tomancak . 2009. “Globally Optimal Stitching of Tiled 3D Microscopic Image Acquisitions.” Bioinformatics 25, no. 11: 1463–1465. 19346324 10.1093/bioinformatics/btp184 PMC2682522

[brb370308-bib-0029] Rowley, C. D. , D. Chitsaz , I. R. Leppert , et al. 2023. “Histological Validation of Myelin‐Sensitive MRI Metrics in the Common Marmoset.” Annual Meeting of the International Society for Magnetic Resonance in Medicine (ISMRM), Toronto, Canada.

[brb370308-bib-0030] Rowley, C. D. , I. R. Leppert , J. S. W. Campbell , et al. 2021. “g‐Ratio in the Common Marmoset: A Comparison Across Different Myelin‐Sensitive MRI Metrics with b‐Tensor Encoded Diffusion.” Annual Meeting of the International Society for Magnetic Resonance in Medicine (ISMRM), Virtual, May 15–20.

[brb370308-bib-0031] Schaeffer, D. J. , L. M. Klassen , Y. Hori , et al. 2022. “An Open Access Resource for Functional Brain Connectivity From Fully Awake Marmosets.” Neuroimage 252: 119030. 35217206 10.1016/j.neuroimage.2022.119030 PMC9048130

[brb370308-bib-0032] Schindelin, J. , I. Arganda‐Carreras , E. Frise , et al. 2012. “Fiji: An Open‐Source Platform for Biological‐Image Analysis.” Nature Methods 9, no. 7: 676–682. 22743772 10.1038/nmeth.2019 PMC3855844

[brb370308-bib-0033] Shimogori, T. , A. Abe , Y. Go , et al. 2018. “Digital Gene Atlas of Neonate Common Marmoset Brain.” Neuroscience Research 128: 1–13. 29111135 10.1016/j.neures.2017.10.009

[brb370308-bib-0034] Sternberger, N. H. , Y. Itoyama , M. W. Kies , and H. D Webster . 1978. “Myelin Basic Protein Demonstrated Immunocytochemically in Oligodendroglia Prior to Myelin Sheath Formation.” PNAS 75, no. 5: 2521–2524. 353815 10.1073/pnas.75.5.2521 PMC392586

[brb370308-bib-0035] Suah, F. B. M. , M. Ahmad , and F. S. Mhamod . 2017. “Effect of Non‐Ionic Surfactants to the Al(III)‐Morin Complex and Its Application in Determination of Al(III) Ions: A Preliminary Study.” Malaysian Journal of Analytical Sciences 21, no. 4: 793–800.

[brb370308-bib-0036] Tardif, S. D. , D. A. Smucny , D. H. Abbott , K. Mansfield , N. Schultz‐Darken , and M. E. Yamamoto . 2003. “Reproduction in Captive Common Marmosets (*Callithrix jacchus*).” Comparative Medicine 53, no. 4: 364–368. 14524412

[brb370308-bib-0037] Todorich, B. , J. M. Pasquini , C. I. Garcia , P. M. Paez , and J. R Connor . 2009. “Oligodendrocytes and Myelination: The Role of Iron.” Glia 57, no. 5: 467–478. 18837051 10.1002/glia.20784

[brb370308-bib-0038] Tokuno, H. , I. Tanaka , Y. Umitsu , T. Akazawa , and Y. Nakamura . 2009. “Web‐Accessible Digital Brain Atlas of the Common Marmoset (*Callithrix jacchus*).” Neuroscience Research 64, no. 1: 128–131. 19428691 10.1016/j.neures.2009.02.003

[brb370308-bib-0039] Weisblum, B. , and E. Haenssler . 1974. “Fluorometric Properties of the Bibenzimidazole Derivative Hoechst 33258, a Fluorescent Probe Specific for AT Concentration in Chromosomal DNA.” Chromosoma 46, no. 3: 255–260. 4136742 10.1007/BF00284881

[brb370308-bib-0040] Yun, J. W. , J. B. Ahn , and B. C. Kang . 2015. “Modeling Parkinson's Disease in the Common Marmoset (*Callithrix jacchus*): Overview of Models, Methods, and Animal Care.” Laboratory Animal Research 31, no. 4: 155–165. 26755918 10.5625/lar.2015.31.4.155 PMC4707143

